# Aqueous Radical Initiated Oxidation of an Organic Monolayer at the Air–Water Interface as a Proxy for Thin Films on Atmospheric Aerosol Studied with Neutron Reflectometry

**DOI:** 10.1021/acs.jpca.3c03846

**Published:** 2023-10-13

**Authors:** Stephanie H. Jones, Martin D. King, Adrian R. Rennie, Andrew D. Ward, Richard A. Campbell, Arwel V. Hughes

**Affiliations:** † Centre of Climate, Ocean and Atmosphere, Department of Earth Sciences, Royal Holloway University of London, Egham, Surrey TW20 0EX, U.K.; ‡ STFC, Central Laser Facility, Research Complex at Harwell, Rutherford Appleton Laboratory, Harwell Oxford, Didcot, Oxfordshire OX11 0FA, U.K.; § Department of Chemistry, Angström Laboratory, Uppsala University, 75121 Uppsala, Sweden; ∥ Institut Laue-Langevin, BP 156, 6, 71 avenue des Martyrs, CS 20156, F-38042 Cedex 9 Grenoble, France; ⊥ ISIS Pulsed Neutron and Muon source, Rutherford Appleton Laboratory, Harwell Oxford, Oxfordshire OX11 0QX, U.K.

## Abstract

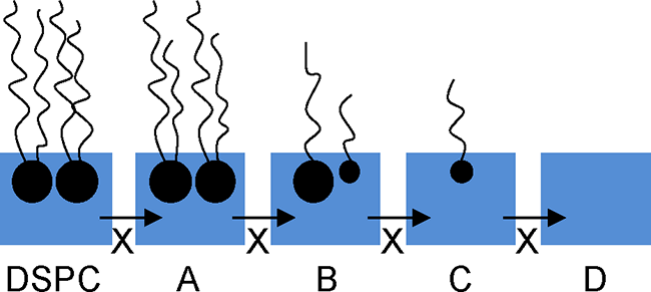

Neutron reflectometry has been used to study the radical initiated oxidation of a monolayer of the lipid 1,2-distearoyl-*sn*-glycero-3-phosphocholine (DSPC) at the air–solution interface by aqueous-phase hydroxyl, sulfate, and nitrate radicals. The oxidation of organic films at the surface of atmospheric aqueous aerosols can influence the optical properties of the aerosol and consequently can impact Earth’s radiative balance and contribute to modern climate change. The amount of material at the air–solution interface was found to decrease on exposure to aqueous-phase radicals which was consistent with a multistep degradation mechanism, i.e., the products of reaction of the DSPC film with aqueous radicals were also surface active. The multistep degradation mechanism suggests that lipid molecules in the thin film degrade to form progressively shorter chain surface active products and several reactive steps are required to remove the film from the air–solution interface. Bimolecular rate constants for oxidation via the aqueous phase OH radical cluster around 10^10^ dm^3^ mol^–1^ s^–1^. Calculations to determine the film lifetime indicate that it will take ∼4–5 days for the film to degrade to 50% of its initial amount in the atmosphere, and therefore attack by aqueous radicals on organic films could be atmospherically important relative to typical atmospheric aerosol lifetimes.

## Introduction

Atmospheric aerosols exist as complex chemical mixtures in the troposphere^1−6^ and are known to influence the Earth’s climate, directly by scattering and absorbing radiation and indirectly through formation of cloud condensation nuclei (CCN) and ice nuclei (IN) and their resultant impact on cloud properties.^7,8^ The aerosol contribution to radiative forcing of the climate remains a major source of uncertainty, and it is therefore necessary to study how aerosol and cloud droplet properties change as a result of common tropospheric reactions such as oxidation.

Thin organic films exist on the surface of aerosols at the air–water interface and the air–solid interface.^9−18^ Marine aerosol, formed via bubble bursting action over the ocean, is a well-known abundant tropospheric aerosol coated with an organic surface active film.^9,10,14^ The coating originates from organic material, including lipids, present in the sea surface microlayer (tens to hundreds of micrometers of the uppermost ocean).^10,19,20^ Lipids that are present in plant waxes and become airborne by wind action are also a terrestrial source of thin organic films on aerosols.^21^ Such organic coatings are believed to form an encapsulating film on the aerosol surface and thus play a central role in aerosol chemistry affecting surface tension and hygroscopic (ability to uptake water) properties.^9−12,22,23^ Films on atmospheric aerosols change their light scattering properties which may change their ability to change the Earth’s albedo as demonstrated by simple 1D radiative-transfer models.^24^ The presence of the film at the aerosol’s interface and its thickness may be more important than the film’s molecular identity.^25^ Atmospheric aerosol is readily oxidized in the atmosphere via a number of gaseous and liquid-phase oxidants,^26,27^ resulting in changes in aerosol chemical, physical, and optical properties.^28^ Thin organic films present on the surface of aerosol may also be readily oxidized, causing changes in coated aerosol properties, such as hygroscopicity, optical properties, and aerosol atmospheric lifetime.^7,29−33^ Several studies have suggested that the oxidation of an organic film at the air–water interface may influence the formation and activation of cloud droplets.^22,30,31,33−36^ As an example, King et al.^33,35^ demonstrated the oxidation of an oleic acid film at the air–water interface as a proxy for an organic coating on aerosol and found that the critical supersaturation required for droplet formation will be lowered as a result.

Atmospheric oxidants exist in both the gaseous and aqueous phase; common gas phase oxidants include ozone,^37,38^ hydroxyl,^39^ and nitrate radicals^40^ and common aqueous phase cloudwater oxidants include hydroxyl, sulfate, and nitrate radicals.^41^ The authors acknowledge the presence of other oxidants such as aqueous ozone and carbonate (CO_3_^•–^) present in terrestrial waters^42^ and emphasize that the current study focuses on processes occurring in cloudwater. A coated aerosol could be oxidized from the gaseous phase above the film, i.e., from the “top-down”,^25,35^ or from the aqueous phase below the film, i.e., from the “bottom-up”. Oxidation from the “bottom-up” has been shown for solid films and radicals generated from the solid phase.^43^ Karagulian et al.^43^ studied the oxidation of a solid monolayer of the lipid 1-oleoyl-2-palmitoyl-*sn*-glycero-3-phosphocholine (OPPC) coated on a mixture of NaNO_2_ and NaCl. OH radicals were formed on illumination of the system, and oxidation of the OPPC film was observed using diffuse reflection infrared Fourier transform spectrometry (DRIFTS) and matrix assisted ionization mass spectrometry (MALDI). The study suggested that similar reactions could also occur for aqueous aerosols coated with an organic film. Jones et al.^44^ used X-ray reflectivity to study the oxidation of atmospheric films using material extracted from atmospheric filter samples at the air–solution interface and conducted experiments from the “bottom-up” using the aqueous phase radicals OH and nitrate radical to demonstrate that the atmospheric film was susceptible to aqueous reaction.

It is known that aqueous cloud processes oxidize thin films, but little is known about the kinetics. Therefore, in order to assess the importance of aqueous-phase oxidation of thin films and to determine the film lifetime with respect to oxidation, the investigation of the aqueous phase oxidation of a well-defined organic film of the lipid 1,2-distearoyl-*sn*-glycero-3-phosphocholine (DSPC) by the common cloudwater aqueous phase radicals, hydroxyl (^•^OH),^45−48^ sulfate (SO_4_^•–^)^27,41^ and nitrate (NO_3_^•^),^27,41,49^ was performed. The reactions were probed by a series of neutron reflectometry experiments using films spread on the surface of solutions in a Teflon trough.

Neutron reflectometry is a surface reflection technique that can provide information about the physical properties of molecular monolayers and reaction kinetics at the air–solution interface.^50−52^ In the present work, neutron reflectometry was used to follow the surface coverage and estimate the thickness of the films at the air–solution interface as they react. Previous neutron reflectometry studies have been successfully conducted on the gas-phase oxidation of atmospherically relevant monolayers at the air–water interface.^25,35,44,53−55^ X-ray reflectivity has also been used to study the susceptibility of real atmospheric films^44^ to oxidation from the gaseous and aqueous phase. The lipid DSPC was chosen for the current study as it is a naturally occurring phosphocholine lipid that forms organic films^56,57^ with a well-known chemical structure and is therefore well suited to kinetic studies.

The following aqueous phase reactions were studied using neutron reflectometry to follow the decay of material (DSPC film and resultant products) at the air–solution interface:
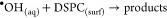
R1
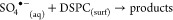
R2
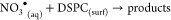
R3where “surf” is used to indicate the air–solution interface.

The oxidative loss of DSPC from the air–solution interface was subsequently kinetically modeled to reproduce the surface coverage as a function of reaction time. A bimolecular rate constant was determined for the loss of surface-active material at the air–solution interface, thus allowing the calculation of a film lifetime with respect to radical oxidation in the atmosphere. Comparison of the film lifetime with typical atmospheric aerosol residence times allows the importance of such aqueous phase radical reactions to be assessed and informs on whether such reactions should be included in atmospheric and aerosol/cloud models.

## Experimental Methods

The section described here includes an introduction to neutron reflectometry and the associated experimental setup used in the neutron reflection experiments. An explanation of aqueous radical generation is provided as well as a description of the calculation of the radical steady-state concentration.

### Neutron Reflectometry

Neutron reflectometry was used to study a monolayer film at the air–solution interface.^50^ Specular neutron reflection allows the determination of the neutron refractive index profile normal to the air–solution interface.^51^ The reflectivity of the film can be modeled and compared to experimental data to obtain information on the amount and thickness of material at the air–solution interface.

Measurements were made at the ISIS neutron source at the Rutherford Appleton Laboratory, Oxfordshire, UK, using the SURF reflectometer^58^ and at the Institut Laue-Langevin (ILL), Grenoble, France, using the FIGARO reflectometer.^59^ At ISIS, neutron reflection was recorded at an angle of 1.5° to the horizontal using a wavelength range of 0.5–6 Å. At the ILL, neutron reflection was recorded at an incident angle of 0.62° to the horizontal with a wavelength range of 2–20 Å and at an incident angle of 3.8° to the horizontal with a wavelength range of 2–30 Å.

The application of the neutron reflection technique to species at the air–water interface has been described by Lu et al.^50^ and therefore will only be described briefly here. Neutron reflectivity, *R*, is measured as a function of the momentum transfer, *Q*, of the neutron^58^ where *Q* is defined as
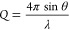
Awhere λ is the neutron wavelength and θ is the incident angle of the neutron beam.

Neutron reflectivity^50^ for a monolayer of deuterated material at the air–water interface is approximately given by
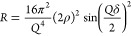
Bwhere δ is the film thickness and ρ is the scattering length density^51^ defined as
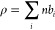
Cwhere *b* is the neutron scattering length of a nucleus and *n* is the number density of that element.^48^ The value of *b* for fully deuterated DSPC (C_44_H_5_NO_8_PD_83_) used in this study is 888.34 fm based on values of *b* for individual elements.^60^ The reflectivity data were normalized using the intensity spectrum of the direct beam of neutrons, and the absolute reflectivity was determined using a scale factor obtained from measuring the reflectivity of pure D_2_O in the trough.

Isotopes of the same element typically have different neutron scattering lengths^60^ where *b* depends on the interaction between the nucleus of an atom and a neutron.^61^ The difference in bound coherent scattering length between hydrogen and deuterium is distinct, *b* = −3.74 fm for hydrogen and *b* = 6.67 fm for deuterium, whereas the chemical properties are the same. Specific chemical moieties of a molecule can be selectively deuterated in order to highlight the deuterated parts by producing a strong contrast between the film, subphase and air.^50^ The subphase is the medium below the film, i.e., the monolayer film to be studied is spread on top of the subphase, a radical precursor solution in the experiments described here. In our study, the reflectivity of the film was increased relative to the air and subphase by using fully deuterated DSPC (C_44_H_5_NO_8_PD_83_) in order to achieve a large scattering length density (ρ = 7.17 × 10^–6^ Å^–2^, calculated from values given by Hollinshead et al.^62^) in comparison to that of the subphase. The subphase had a scattering length density of zero (effectively the same as the air above the film) and was therefore null reflecting. A solution of so-called null reflecting water (NRW) consists of D_2_O and H_2_O in the volume proportion 8.1% and 91.9%, respectively. In the current experiment, aqueous radicals were generated photolytically from the subphase, and consequently, the volume ratio of D_2_O and H_2_O in the subphase was slightly adjusted to account for the scattering length density of the radical precursor chemicals to achieve an overall value of zero scattering length density. A monolayer of fully deuterated DSPC was spread on top of the null reflecting radical precursor subphase contained in the trough, and the specular neutron reflection signal was dominated by reflection from the monolayer film. Neutron reflectometry measures the combined amount of reactant and any insoluble, involatile, surface-active reaction product that remains at the air–solution interface, meaning it is a convolution of the remaining reactants and any products that have not yet dissolved or evaporated.

In the current study, neutron reflectivity as a function of *Q* was typically recorded continuously in intervals of 300 or 900 s (i.e., time to record one reflectivity profile *R* vs *Q*). Each kinetic run monitoring reactions R1–R3 was ∼50 000 s in duration. The experimental reflectivity versus the momentum transfer was modeled with a single uniform layer using the Abelès formalism,^63^ which allows determination of the scattering length density and thickness of the deuterated material at the air–solution interface.

Scattering length density ρ, and film thickness δ, can be related to the scattering length *b*, of a molecule of a film composed entirely of the same molecule, and its surface coverage, Γ, by^35^
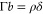
DHowever, the scattering length, *b*, is not constant in a reacting system where the molecule does not leave the air–solution interface, and a mixture of different product molecules may also be present at the air–solution interface with different neutron scattering lengths, contributing to the overall neutron reflectivity. Therefore, the scattering length per unit area, ρδ, of all of the material at the air–solution interface is kinetically followed as a function of time, ρ_*t*_δ_*t*_. As kinetics is the focus of the present study, the quantity
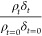
Ewas followed as a function of time, where ρ_*t*=0_δ_*t*=0_ is the product of the scattering length density and film thickness at time zero.

As will be detailed in the Multistep Degradation Kinetics section, to fit the variation of *equation E* to individual molecular components making up the film with time, the weighted sum of the individual products of scattering length, *b*, and surface coverage, Γ, for each component need to be considered.

MOTOFIT^64^ was used to reproduce the experimental reflectivity data.

### Experimental Setup

A custom PTFE trough with an area of of 240 × 70 mm^2^ and a liquid depth of approximately 3.5 mm was used in the study. UV lamps were suspended above the trough along its length to photolytically generate the radicals and provide an even illumination of the air–solution interface. Two types of UV lamps were used, black lamps centered at wavelength ∼360 nm (UVP) to generate SO_4_^•–^ and NO_3_^•^ radicals and germicidal lamps that provided a significant output at ∼254 nm (UVP) to generate ^•^OH radicals. In both cases, the bulbs were situated in a retroreflector within the lamp body at a fixed height above the air–solution interface. The trough and lamps were loosely surrounded by a Tedlar plastic film to keep the interface free from dust.

A monolayer of fully deuterated DSPC (C_44_H_5_NO_8_PD_83_), >99% atom purity, from Avanti Polar Lipids Inc. was spread on the surface of the radical precursor aqueous subphase as a 1 mg mL^–1^ solution in chloroform (Sigma-Aldrich, 0.5–1% ethanol as stabilizer) using a glass microliter syringe. In each experiment, 40 μL was typically added to the air–solution interface. Previously it had been determined that 40 μL provided a surface pressure of ∼20 mN m^–1^ on the trough used in these experiments. Surface pressure was not measured during the neutron studies because UV radiation degrades the tensiometer and would have blocked the neutron beam on the small trough used to achieve even illumination from the photolysis lamp. Neutron reflectivity as a function of momentum transfer, *Q*, was recorded before and during photolysis of the subphase to monitor the change in the DSPC monolayer.^58,59^

### Radical Generation

Aqueous radicals were photolytically produced from the subphase using aqueous phase photochemistry.^26,65−68^ Hydroxyl radicals were generated by photolysis of a 0.03 mol dm^–3^ aqueous solution of KNO_3_ by the method of Mack and Bolton^65^ using two UV bulbs centered at ∼254 nm (UVP) and placed at a height of ∼83 mm above the trough. A clean well-established method of producing hydroxyl radicals as demonstrated by Karagulian et al.^43^
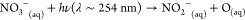
R4
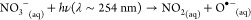
R5
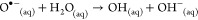
R6

Sulfate radical anions were generated by photolysis of a 0.03 mol dm^–3^ aqueous solution of K_2_S_2_O_8_^66^ using four black bulbs centered at ∼360 nm (UVP) at a height of 128 mm above the trough.
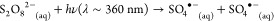
R7

Nitrate radicals were generated by photolysis of an aqueous solution subphase containing 0.03 mol dm^–3^ K_2_S_2_O_8_ and 0.1 mol dm^–3^ KNO_3_ by the method of Herrmann,^26^ using four black bulbs centered at ∼360 nm (UVP) and placed at a height of 128 mm above the trough at ISIS and 115 mm above the trough at the ILL. The height of the lamps was used to control the flux of photons and associated rate of reaction. Photolysis of S_2_O_8_^2–^ yielded two SO_4_^•–^ radicals as detailed in eq R7 and subsequent titration of SO_4_^•–^ with an excess of NO_3_^–^ resulted in the production of the nitrate radical, NO_3_^•^ as described by Herrmann.^26^
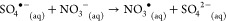
R8

The UV lamps used to initiate photolysis were switched on and off remotely and produced a stable irradiance after 3 min.^69^ The concentration of the aqueous radical precursor was chosen to maintain a constant steady-state radical concentration throughout the entire kinetic run. The focus is on the radicals generated, not the radical precursors used, with the only requirement being that the precursors do not interfere with the reaction.

Two sets of control experiments were performed, “dark controls” conducted without UV light on a monolayer of DSPC on the aqueous solution subphases of 0.03 mol dm^–3^ KNO_3_, 0.03 mol dm^–3^ K_2_S_2_O_8_ with 0.1 mol dm^–3^ KNO_3_, and 0.03 mol dm^–3^ K_2_S_2_O_8_ and “light or photolysis controls” conducted with UV light on a monolayer of DSPC on null reflecting water with no radical precursors present.

### Kinetic Modeling

Two kinetic models were developed in the work presented here: a multistep degradation of material at the air–solution interface and a steady-state analysis of the radical concentration. The secondary chemistry following photolysis required determination of the steady-state concentrations of hydroxyl radicals and sulfate radical anions by kinetic modeling as described below. It was not possible to attempt the same analysis and modeling with the nitrate radical as the secondary chemistry is not accurately known. Note that the steady-state analysis of the radical concentration (^•^OH, NO_3_^•^ or SO_4_^•–^) is strictly a determination of the “bulk” concentration of radical. The very large excess of the photolabile radical precursor (NO_3_^–^ and S_2_O_8_^2–^) should prevent a reduction in the radical concentration near the air–solution interface owing to reaction.

### Determination of Radical Steady-State Concentrations

The steady-state concentrations were measured in a series of off-line experiments. The loss of radical precursor was measured as a function of photolysis time to determine the photolysis rate constant, *J*, for eqs R4, R5, and R7 offline. The offline experimental setup was the same as that used for the neutron reflectometry experiments. Kinetic modeling was required because the photolysis rates of the NO_3_^–^ and S_2_O_8_^2–^ anions were unknown. During the neutron reflectometry experiments, the concentration of radical precursors (NO_3_^–^ and S_2_O_8_^2–^) was kept large to maintain an effectively constant concentration of radical precursor and thus a constant concentration of radical in steady-state concentration throughout the reaction. However, in order to calculate the photolysis rate constant for NO_3_^–^ and S_2_O_8_^2–^ the precursor solutions were photolyzed at smaller concentrations of 0.0003 mol dm^–3^ KNO_3_ aqueous solution and 0.0003 mol dm^–3^ K_2_S_2_O_8_ aqueous solution to observe and model the loss of radical precursor. Loss of KNO_3_ or K_2_S_2_O_8_ was followed by UV–visible spectrometry for up to 10 h for K_2_S_2_O_8_ and up to 16.5 h for KNO_3_. The following photolysis reaction schemes were kinetically modeled using a Backward Differentiation Formula (BDF) method to handle stiff systems,^70−72^ and the value of the appropriate photolysis rate constants, *J*, was varied until the modeled temporal variation of the concentrations of NO_3_^–^, NO_2_^–^ and S_2_O_8_^2–^ fitted the experimentally determined temporal profile of the concentrations. Rate constants for the reactions in the photolysis schemes were obtained from the literature where possible, and the following rate constants were determined from kinetic modeling: *J*_4_, *J*_5_, *J*_7_, *J*_11_, and *k*_12_.

Hydroxyl radicals are produced from the photolysis of nitrate from the following photochemistry:
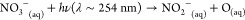
R4*J*_4_ = 3.5 × 10^-6^ s^–1^
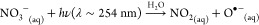
R5*J*_5_ = 3.5 × 10^-6^ s^–1^
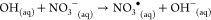
R9*k*_9_ = 1.3 × 10^8^ M^–1^ s^–1^^73^
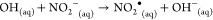
R10*k*_10_ = 6.0 × 10^9^ M^–1^ s^–1^^74^
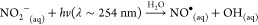
R11*J*_11_ = 5.0 × 10^–5^ s^–1^
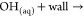
R12*k*_12_ = 1 s^–1^
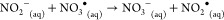
R13*k*_13_ = 6.0 × 10^9^ M^–1^ s^–1^^74^
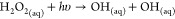
R14*k*_14_ = 0
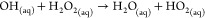
R15*k*_15_ = 4.2 × 10^7^ M^–1^ s^–1^^75^
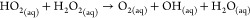
R16*k*_16_ = 0.5 M^–1^ s^–1^^75^
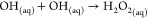
R17*k*_17_ = 6 × 10^9^ M^–1^ s^–1^^75^
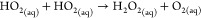
R18*k*_18_ = 980000 M^–1^ s^–1^^75^
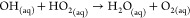
R19*k*_19_ = 1.0 × 10^10^ M^–1^ s^–1^^75^
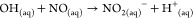
R20*k*_20_ = 1.5 × 10^10^ M^–1^ s^–1^^65^
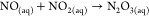
R21*k*_21_ = 1.1 × 10^9^ M^–1^ s^–1^^65^
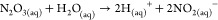
R22*k*_22_ = 530 s^–1^^65^
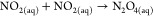
R23*k*_23_ = 4.5 × 10^8^ M^–1^ s^–1^^65^
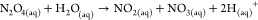
R24*k*_24_ = 1000 s^–1^^65^
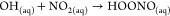
R25*k*_25_ = 1.3 × 10^9^ M^–1^ s^–1^^65^
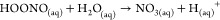
R26*k*_26_ = 1.4 s^–1^^65^

The concentrations of NO_3_^–^ and NO_2_^–^ anions were measured by UV–visible spectrometry over the wavelength range ∼200–500 nm in a long path length liquid cell (17.5 cm) for a series of photolysis times from 2 h up to 16.5 h. The steady-state concentration of ^•^OH radicals was then determined from kinetically modeling the above reactions (reactions R4, R5, and R9–R26) as a series of first-order differential equations and varying *J*_4_, *J*_5_, *J*_11_, and *k*_12_ to obtain a good fit between experimental and modeled [NO_3_^–^] and [NO_2_^–^] with time. The hydroxyl radical steady-state concentration was calculated from the precursor concentration used in the neutron experiments, 0.03 mol dm^–3^, using the kinetic model as ∼1 × 10^–14^ mol dm^–3^.

The photolysis of persulfate, K_2_S_2_O_8_ in aqueous solution leading to the production of sulfate radical anions derives from the following photochemistry:^73^
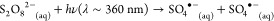
R7*J*_7_ = 1.32 × 10^–5^ s^–1^
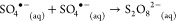
R27*k*_27_ = 6.1 × 10^8^ M^–1^ s^–1^^66^
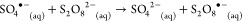
R28*k*_28_ = 5.5× 10^5^ M^–1^ s^–1^^66^
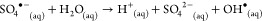
R29*k*_29_[H_2_O] = 460 s^–1^^66^

Owing to the weak absorption of the SO_4_^2–^ anion, the decrease in concentration of S_2_O_8_^2–^ was followed instead of the SO_4_^2–^ anion as a function of photolysis time by UV–visible spectrometry over the wavelength range ∼200–300 nm. The steady-state concentration of SO_4_^•–^ radicals was then determined from kinetically modeling the above reactions (reactions R7 and R27–R29) as a series of first-order differential equations and varying *J*_7_. Similarly to steady-state [OH], denoted as [OH]_ss_, once the photolysis rate constant, *J*_7_, had been determined, the initial persulfate concentration (0.03 mol dm^–3^) was used to calculate the sulfate radical steady-state concentration in the neutron experiments as 4.7 × 10^–11^ mol dm^–3^.

It was noted that light attenuation by the water or aqueous radical precursor solution could occur at 254 or 360 nm; however, light attenuation was found not to be an issue following an e-folding depth calculation as detailed in the Supporting Information (Table S2).

## Results and Discussion

**Figure 1:**
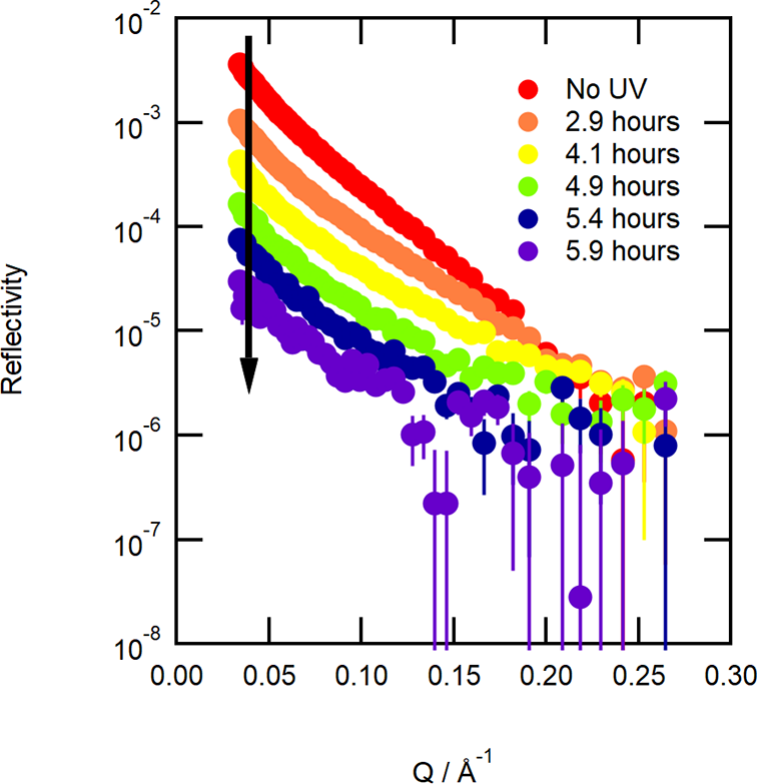
Typical neutron reflectivity profiles for a monolayer film of fully deuterated DSPC at the air–solution interface on exposure to the aqueous phase hydroxyl radical. The reflectivity of the film prior to exposure to the hydroxyl radical is the largest (red circles), and as the UV lights are switched on and the hydroxyl radical is generated, the reflectivity decreases in the direction of the black arrow to the purple circles (lowest reflectivity). Data were recorded on FIGARO.

Figure 1 shows a typical decay in the neutron reflectivity profile with time as a monolayer film of DSPC at the air–solution interface is exposed to the aqueous phase hydroxyl radical. Note that not all reflectivity profiles are shown for clarity.

### Determination of Steady-State Radical Concentrations

The steady-state concentrations of reactant radicals listed in Table 1 were calculated by fitting the temporal decay of NO_3_^–^, NO_2_^–^, and S_2_O_8_^2–^ as described in the determination of radical steady-state concentration in the experimental section and are shown in Figures S1 and S2 in the Supporting Information. Determination of the steady-state concentration was the largest source of uncertainty in the analysis. The concentration of OH radicals was sensitive to the photolysis rate constants (eqs R4, R5, and R11). Varying the value of these rate constants (eqs R4, R5, and R11) by a factor of ±40% led to a corresponding change in OH radical concentration of ±30%. Uncertainty analysis involved adjustment of the photolysis rate constants by fixed amounts and determination of the resultant steady-state concentration. From the analysis, both the hydroxyl and sulfate steady-state concentrations were determined to be within 40% of the values given in Table 1. Figure S3 shows the ±40% fit of the photolysis rate constants for the hydroxyl radical as well as ±20, 60 and 80%. A ±40% variation in the photolysis rate constants corresponds to a ±30% variation in the OH steady-state concentration. The determination of the hydroxyl radical concentration was performed in a series of offline experiments. The apparatus, including lamps, scaffolding, trough, and chemicals, was exactly the same as the experimental apparatus used for determination of the kinetics of oxidation of the lipid. The only difference was that the concentration of radical precursor is lower in the offline experiments to allow the radical precursor concentration (nitrate anion or peroxydisulfate ion) to decrease and be measured in order to determine the photolysis rate coefficients of the precursor species. In some aspects the offline steady-state determination experiments are similar to a traditional actinometry experiment^76^ but using the compound of photolysis as the actinometer.

**Table 1:** Determined Values of Pseudo-First-Order and Bimolecular Rate Constants and Aqueous Steady-State Radical Concentrations for the Multistep Degradation Mechanism for the Aqueous Phase Hydroxyl and Sulfate Radical Systems^a^

Radical reaction	*k*′ (10^–4^ s^–1^)	Steady-state radical concentration (mol dm^–3^)	*k* (mol^–1^ dm^3^ s^–1^)^b^
^•^OH R36	3.5	1 × 10^–14^	3.5 × 10^10^
^•^OH R37	2.1	1 × 10^–14^	2.1 × 10^10^
^•^OH R38	2.3	1 × 10^–14^	2.3 × 10^10^
^•^OH R39	1.9	1 × 10^–14^	1.9 × 10^10^
SO_4_^•–^R36	2.22 ± 0.56	4.7 × 10^–11^	(3.77 ± 2.15) × 10^6^
SO_4_^•–^R37	1.85 ± 0.47	4.7 × 10^–11^	(3.93 ± 0.99) × 10^6^
SO_4_^•–^R38	1.85 ± 0.47	4.7 × 10^–11^	(3.93 ± 0.99) × 10^6^
SO_4_^•–^R39	1.85 ± 0.47	4.7 × 10^–11^	(3.93 ± 0.99) × 10^6^

a Average of the pseudo-first-order (reactions R36–R39) and bimolecular rate constants (determined using eq R44 and the modeled aqueous radical steady-state concentrations) for each degradation step in the multistep degradation mechanism for ^•^OH and SO_4_^•–^, respectively. Two experiments were conducted using ^•^OH, and hence no standard deviation is reported.

b Note the diffusion limited bimolecular rate constant can be crudely estimated from *k* = 8*RT*/(3η) ≈ 6 × 10^9^ M^–1^ s^–1^ assuming a temperature of 298 K and a viscosity of water, η, of 0.001 kg m^–1^ s^–1^.^83^

### Multistep Degradation Kinetics

It is useful to describe here the kinetic scheme used to model the decay of relative scattering length with the time of the organic layer at the air–solution interface. A multistep degradation mechanism^25,77^ was fitted to the experimentally determined decrease in surface coverage of material with time at the air–solution interface. The characteristic temporal profile and lack of exponential decay led to consideration of a multistep degradation mechanism. The following mechanism is proposed:
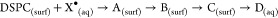
R30where DSPC_(surf)_ is a monolayer of DSPC at the air–solution interface; X^•^_(aq)_ is an aqueous radical oxidant (either ^•^OH, NO_3_^•^, or SO_4_^•–^); A_(surf)_, B_(surf)_ and C_(surf)_ are surface active deuterated products at the air–solution interface; and D_(aq)_ is a deuterated product that is not surface-active, i.e., A, B, and C reside at the air–solution interface whereas product D partitions irreversibly to either the gas-phase or the aqueous subphase. Experimentally determined surface coverage was defined as the weighted sum of the surface coverage of all proposed deuterated product species at the air–solution interface including the original DSPC film and any other deuterated surface-active products that may have formed from reaction with the radical species, i.e., products A–C. Each proposed surface-active product (A–C) has a scattering length density that is consistent with oxidation removing parts of the original DSPC molecule (α, β, and γ in eq R31). It should be noted that the “true” mechanism will be more complicated as the number of products from the initial radical attack will be large. As such A, B, C, and D represent an ensemble of products, which have the typical scattering length density represented in eq R31. Thus, the total surface coverage at the air–solution interface determined by neutron reflection was defined as the weighted sum of Γ_DSPC_, Γ_A_, Γ_B_, and Γ_C_:
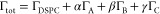
R31where α, β, and γ are the weightings relating to the scattering length density, ρ, of each surface-active decay product, i.e.,
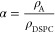
R32
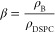
R33
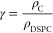
R34

Values of α, β, and γ were empirically taken as 0.95, 0.83, and 0.41 to represent stylized decay steps. The number of degradation steps and the values of α, β and γ were the result of trial and error during the fitting process and were selected to achieve a good fit between the model and experimental data.^78^
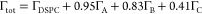
R35

As detailed below and in part 2 of the Supporting Information, a sensitivity study was performed on the number degradation steps and the values of α, β, and γ to ensure the use of optimum values that best described the temporal profile of the deuterated material at the interface. The experimentally determined surface coverage (eq E) as a function of reaction time was fitted to the following degradation scheme:
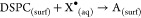
R36
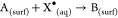
R37
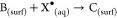
R38
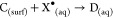
R39where X = OH or SO_4_^•–^, also a result of trial and error to obtain good agreement between the modeled and calculated temporal profiles of deuterated material at the air–solution interface.^78^

Reactions R36–R39 are modeled as a single reaction step with the OH radical. The single reaction represents significant well-known secondary chemistry^79^ and may include autoxidation.^80−82^ The determination of the value of rate constants for reactions R36–R39 may then represent an overestimation depending on the chain length^78^ of the secondary chemical reactions. However, the secondary chemistry of the oxidation of single molecule thick films at the air–water interface is likely different from reactions within bulk organic particles of an aerosol. Unfortunately, the technique to study the kinetics described, while excellent for studies of monomolecular films at interfaces, does not produce the detailed chemical speciation to enable the secondary chemistry to be elucidated. Reactions of DSPC, A, B, and C with the OH radical (reactions R36–R39) may not be replaced by separate autoxidation events as demonstrated by switching off the photolysis lamp during reaction and noting a halt in the loss of material (neutron scattering length per unit area) from the air–solution interface. Reactions R36–R39 therefore suggest the removal of a molecule from a film of DSPC and its reaction products from the air–solution interface, consistent with approximately four radical attacks from photolytically generated radicals. The reactions R36–R39 have the following rate laws:^78^
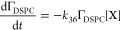
R40
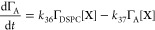
R41
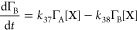
R42
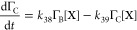
R43

The concentration of the radicals [X] in the vicinity of the air–solution interface are in steady-state during the reaction, i.e., d[X]/d*t* = 0, thus a series of pseudo-first-order rate constants, *k′*, can be defined as:
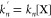
R44where *n* = 36, 37, 38, and 39 and eqs R40–R43 become:
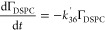
R45
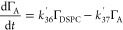
R46
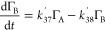
R47
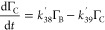
R48Equations R32–R35 were kinetically modeled using a Runge–Kutta method,^70^ and values of the pseudo-first-order rate constants were varied until the modeled temporal variation of the total surface coverage, Γ_tot_, eq R35 fitted the experimentally determined temporal profile of surface coverage for each neutron experiment. The fitting of the kinetic eqs R36—R39 to the decay of  versus time, by kinetic modeling a series of first-order differential rate laws is dependent on the number of degradation steps and the values of α, β and γ. A sensitivity study in part 2 of the Supporting Information describes how a chi-squared fitting parameter was used as a metric of the goodness of fit between the modeled and experimentally derived data for  versus time as the number of degradation steps and values of α, β and γ were varied. While the sensitivity study does not explore the entirety of a large parameter space, it gives confidence that the values of α, β, and γ provide a good fit and that the minimum number of degradation steps are included. Note in the above analysis that the very slow diffusion of oxidized organic materials from the interface has not been considered, (diffusion on the time scale of tens of minutes to move 2.5 nm^62^). To estimate the possibility of such a process would require repeating the experiments, stopping the oxidation (by switching off the photolysis lamps) at periodic parts of the reaction, and observing/measuring any further loss of material.

**Figure 2:**
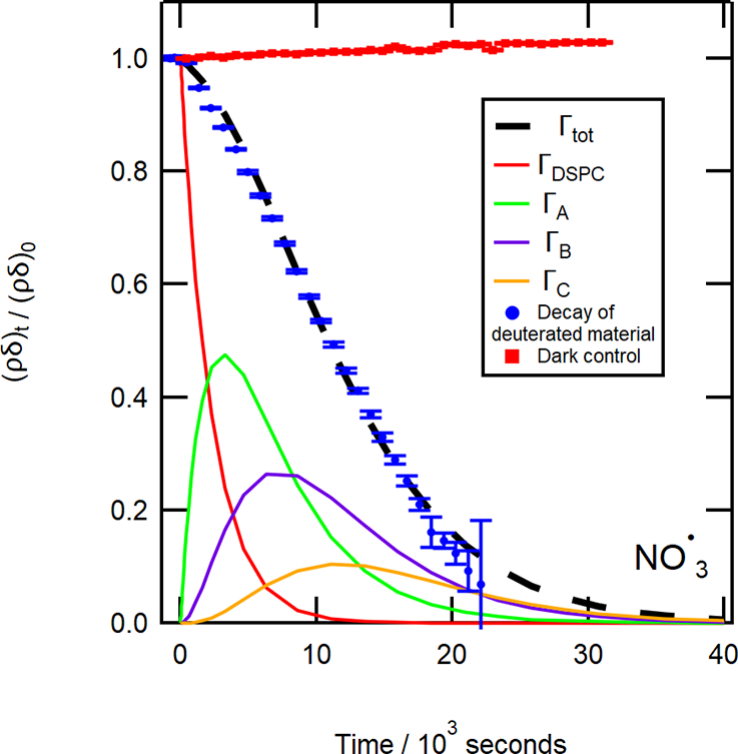
Experimental decay in relative surface coverage due to NO_3_^•^ radicals is shown as blue circles. The modeled fit of the degradation mechanism to the experimental data is shown as the dashed black line. The remaining curves represent the decay of the initial DSPC film (red line) and the build-up and decay of the individual reaction products, A, B, and C. A dark control (no light, red squares), confirms that the reaction required the presence of UV light. It is thought that the gentle rise may be related to changes in the meniscus of the liquid surface as the reaction progressed. Data were recorded on FIGARO.

**Figure 3:**
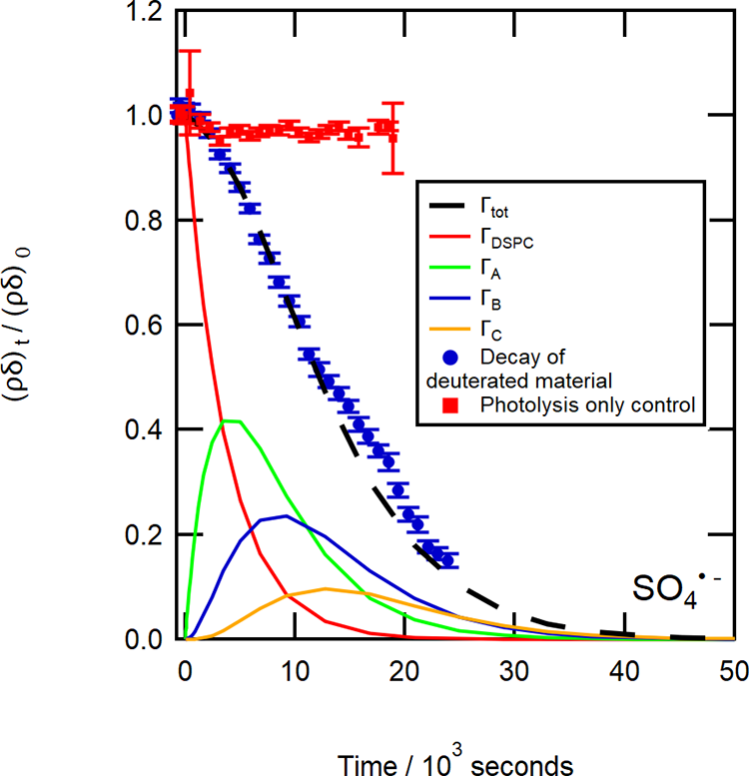
Experimental decay in relative surface coverage due to SO_4_^•–^ radicals is shown as blue circles. The modeled fit of the degradation mechanism to the experimental data is shown as the dashed black line. The remaining curves represent the decay of the initial DSPC film (red line) and the build-up and decay of the individual reaction products. A photolysis control (∼360 nm light, red squares) confirms minimal degradation of the film occurs owing to the presence of UV light. Data were recorded on SURF.

**Figure 4:**
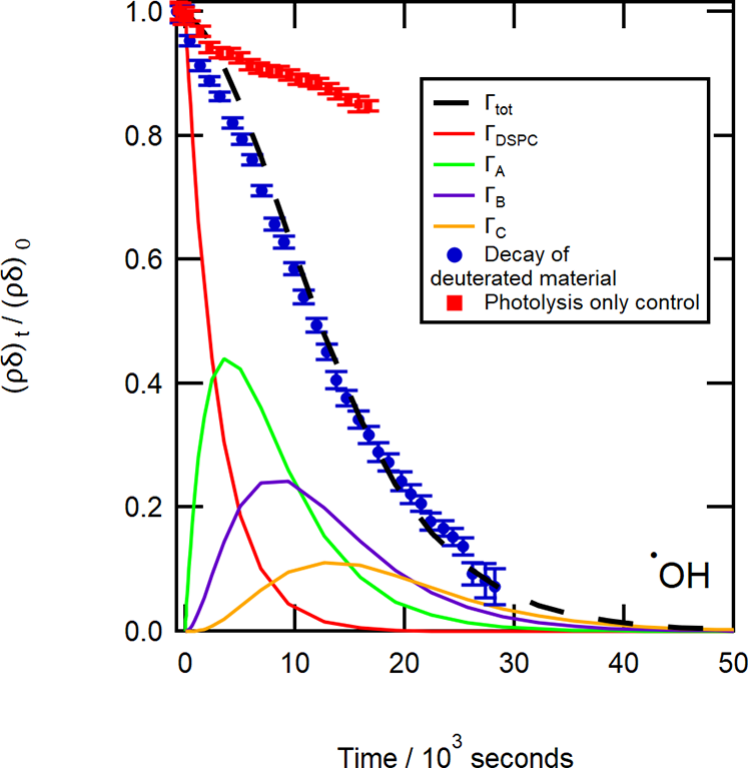
Experimental decay in relative surface coverage due to ^•^OH radicals is shown as blue circles. The modeled fit of the degradation mechanism to the experimental data is shown as a dashed black line. The remaining curves show the decrease in relative surface coverage of a monolayer film of DSPC (red) and the build-up and decrease in relative surface coverage of the reaction products. A photolysis control (∼254 nm light, red squares), confirms some degradation of the film does occur owing to the presence of UV light. Data were recorded on SURF.

Figures 2, 3, and 4 show the experimental decays ( versus time) of a monolayer film of DSPC on exposure to the aqueous phase radicals NO_3_^•^, SO_4_^•–^, and ^•^OH, respectively. The experimental data are reproduced by calculation of eq R35 by varying the values of *k*_36_^′^–*k*_39_^′^ using kinetic modeling. Table 1 highlights the modeled values of *k*′ for each degradation step for each radical system (^•^OH and SO_4_^•–^).

Figures 2, 3, and 4 demonstrate a key result that the surface coverage of material at the air–solution interface can follow a multistep degradation mechanism.^78^ Individual amounts of the modeled surface-active degradation products A, B, and C are also shown in Figures 2, 3, and 4 as well as control runs, which emphasize the difference between degradation and no reaction. Figure 2 includes a dark control, a monolayer film of DSPC on a radical precursor subphase with no light source, i.e., no photolysis. Figures 3 and 4 both show light controls, i.e., photolysis only, for a monolayer film of DSPC on NRW with UV light centered at ∼360 and 254 nm, respectively.

A simple exponential decay has been fitted to the photolysis control (red squares) in Figure 3, which is indicative of a very slow decay in relative surface coverage with time. A decay of ∼4% in relative surface coverage in ∼5.3 h is attributed to a small degradation owing to the presence of UV light only (centered at ∼360 nm) demonstrating that UV radiation alone is not responsible for the loss of material from the interface.

Figure 4 shows the decrease on exposure to the aqueous hydroxyl radical. A ∼71% decrease in the relative surface coverage was observed after 5 h of photolysis (∼254 nm). As in Figure 3, there was a slight decrease in the amount of material for the photolysis control (red squares), so an exponential function was fitted to the decay. The exponential function indicated a ∼15% decay in the initial relative surface coverage during ∼5 h of exposure to UV light (∼254 nm), which was a greater decay than that observed for the light control (∼360 nm) in Figure 3. Comparing the decay from the light control to the decay observed for the radical reaction after 5 h of photolysis, the amount of deuterated material at the air–solution interface had decayed to ∼64% of its initial value.

The pseudo-first-order and bimolecular rate constants reported in Table 1 are averaged for each degradation step for all of the experiments conducted using the aqueous sulfate radical. No uncertainties are reported for the hydroxyl system, as only two experiments were successful and neutron beam time is a very limited resource. All the pseudo-first-order rate constants for each radical system were also varied by factors of 5%, and the resultant fit through the experimental data points assessed by inspection. Variation of *k*′ that provided a reasonable fit by eye was ±5% for the hydroxyl system, ±10% for the sulfate system, and ±20% for the nitrate system. For the nitrate system, a further sensitivity analysis was performed; each pseudo-first-order rate constant was varied individually by 10% and the remaining rate constants were subsequently adjusted to maintain a good fit through the data points. From variation of individual pseudo-first-order rate constants, it was deemed that no one rate constant was more or less significant in determining the reaction profile.

The diffusion of OH radicals to the interface was not considered to be a slow process relative to the rate of loss of OH radicals owing to reaction with the film. Characteristic time scales for diffusion and reaction can be estimated from simple formulas in Finlayson-Pitts and Pitts.^84^ The characteristic time for transport of OH radicals to the interface is , where *d* = 2.5 nm (the approximate size of the DSPC molecule at the air–water interface^62^) and an estimate of the diffusion coefficient *D* = 2.8 × 10^–9^ m^2^ s^–1^^85^ gives ∼0.2 ns. The characteristic reaction time is  where *k* = 3.5 × 10^–4^ s^–1^ taken from Table 1 which gives a value with respect to reaction at the interface as ∼3000 s. A detailed mass-transfer calculation or multilayer diffusion model as described elsewhere^86,87^ is thus not considered. The photolytic source of OH radicals from an enormous excess of nitrate anions was chosen to prevent a diffusive concentration profile from building up to the interface over the course of the long reaction time.

### Bimolecular Rate Constants

The bimolecular rate constants determined for each step in the degradation mechanism (eqs R36–R39) of a DSPC film initiated by aqueous phase ^•^OH radical attack are larger than the bimolecular rate constants determined for oxidation initiated by aqueous phase sulfate radical attack. Comparable values of bimolecular rate constants were determined in recent studies for aqueous phase ^•^OH radical reactions with *cis*-pinonic acid (∼3–4 × 10^9^ M^–1^ s^–1^) and substituted phenols (on order of 10^9^ M^–1^ s^–1^ to 10^10^ M^–1^ s^–1^ depending on the specific phenol and the pH).^88−90^ The subsequent bimolecular rate constants for each step of the degradation mechanism are similar to each other, which is what is expected given that A, B, and C will have similar reactivity toward OH radical, i.e., the radical abstracts a deuterium from DPSC, A, B, and C.

Note that owing to the deuteration of the DSPC film for the neutron reflection technique, there will be a potential kinetic isotope effect on the values of the rate constants. Statistical mechanics has shown the kinetic isotope effect to be up to a factor of 7.2 for hydrogen abstraction,^78^ but the kinetic isotope effect is generally a factor of 6.1 for breaking a C–H bond.^78^ The rate constants have not been corrected for the kinetic isotope effect, and thus, the values in Table 1 represent a slight overestimation for atmospheric studies where the molecules would be predominately undeuterated. Note that a small proportion of our OH radicals will actually be OD radicals. The kinetic isotope effect of having some OD radicals is not considered important.

### Comparison to Other Literature Studies

There have been several studies on the oxidation of thin films by gaseous phase oxidants, e.g. refs (25,35,53−55,91−94). Few studies have focused on oxidation of thin films from the aqueous phase;^44^ however, an increasing number of recent studies focus on aqueous phase oxidation in the bulk^88,89,95−100,90,101^ and in particular by the aqueous phase OH radical.^88−90,95,96,99−101^ Other relevant studies include that of Sarang et al.,^97^ who considered aqueous phase oxidation of so-called “Green Leaf Volatiles”, unsaturated oxygenated hydrocarbons (volatile species emitted from plants that can partition into water), by ^•^OH, SO_4_^•–^, and NO_3_^•^, and that of Tran et al.,^98^ who studied oxidation by the aqueous phase SO_4_^•–^ of a number of organic aerosol compounds including isoprene related compounds, lactic and pyruvic acid.

The second-order rate constants determined in the current study for oxidation by aqueous phase ^•^OH are on the order of 10^10^ M^–1^ s^–1^ and are comparable to other studies of aqueous phase ^•^OH bimolecular constants.^88−90^ Relevant to this study is the study by Witkowski and Gierczak^88^ who determined rate constants on the order 10^9^ M^–1^ s^–1^ for reaction of aqueous phase ^•^OH and cis-pinonic acid. Similar values of bimolecular rate constants to those presented in Table 1 were also determined for the aqueous phase ^•^OH oxidation of substituted phenols.^89,90^ Arciva et al.^90^ determined second-order rate constants on the order of 10^9^–10^10^ M^–1^ s^–1^ for the reaction of highly substituted phenols with the aqueous phase ^•^OH, and He et al.^89^ determined rate constants on the order of 10^9^ M^–1^ s^–1^ for reaction of aqueous phase ^•^OH with methoxyphenolic compounds. Additionally, such reactions have been suggested as an important source of aqueous secondary organic aerosol, SOA.^90,102^

As discussed in the Introduction, Karagulian et al.^43^ studied oxidation from the “bottom up” using OH radicals to oxidize a lipid, 1-oleoyl-2-palmitoyl-*sn*-glycero-3-phosphocholine (OPPC), coating on a solid mixture of NaCl and NaNO_2_ particles. The chemistry observed in the work presented here, i.e., oxidation of a lipid from below in the aqueous phase, supports their hypothesis that such reactions could also occur on aqueous droplets coated in an organic film.

The aqueous phase oxidation temporal profiles of DSPC shown here (Figures 2–4) match those of DSPC and gas phase OH radicals,^25^ suggesting that DSPC reacting with OH from the gaseous phase and the aqueous phase have similar chemical mechanisms. Further comparison of the kinetic profile observed in the current study with those of real film material abstracted from the atmosphere to the aqueous-phase OH and nitrate radical^44^ also indicates a similar temporal profile. Thus, aqueous phase oxidation may preferentially proceed via a multistep degradation mechanism as described in the current work, but more studies on real atmospheric samples are required to confirm this. Additionally, the mechanism may be used to describe the aqueous phase radical induced oxidation of other saturated lipids, including those with different head groups.

### Atmospheric Implications

The lifetime of a monomolecular film of DSPC at the air–solution interface with respect to aqueous phase ^•^OH radical attack was estimated to be ∼20 h using the following equation.
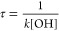
R49where [OH] is an atmospheric steady state concentration of aqueous ^•^OH radicals given as 3.80 × 10^–16^ mol dm^–3^.^103^ Typical atmospheric concentrations for NO_3_^•^ and SO_4_^•–^ are not readily available; therefore, the lifetime of DSPC with respect to NO_3_^•^ and SO_4_^•–^ was not calculated.

The lifetime of DSPC is interesting because it provides a measure of the importance of a lipid coating on atmospheric aerosol. A long-lived lipid (>4–10 days^3^) may not need to be considered in atmospheric oxidation models suggesting that it should be readily found in atmospheric filter samples. The short atmospheric lifetime (<4–10 days^3^) calculated above would suggest that the oxidation chemistry described here is important for atmospheric oxidation modeling. The amount of DSPC measured in atmospheric filters may be less than that released to the environment, as the lifetime is short enough for DSPC to have been partially removed in the atmosphere before being captured on a filter. The lifetime calculated in eq R49 is for DSPC. However, the work presented here has shown that products of reaction R1 remain at the air–solution interface and it is this product film, not just the DSPC, that affects light scattering and evaporation as well as other important processes of atmospheric aerosols. Thus, determination of the film lifetime may be more important than the DSPC lifetime, i.e., the time to remove DSPC and all its reaction products that favor the air–solution interface.

**Figure 5:**
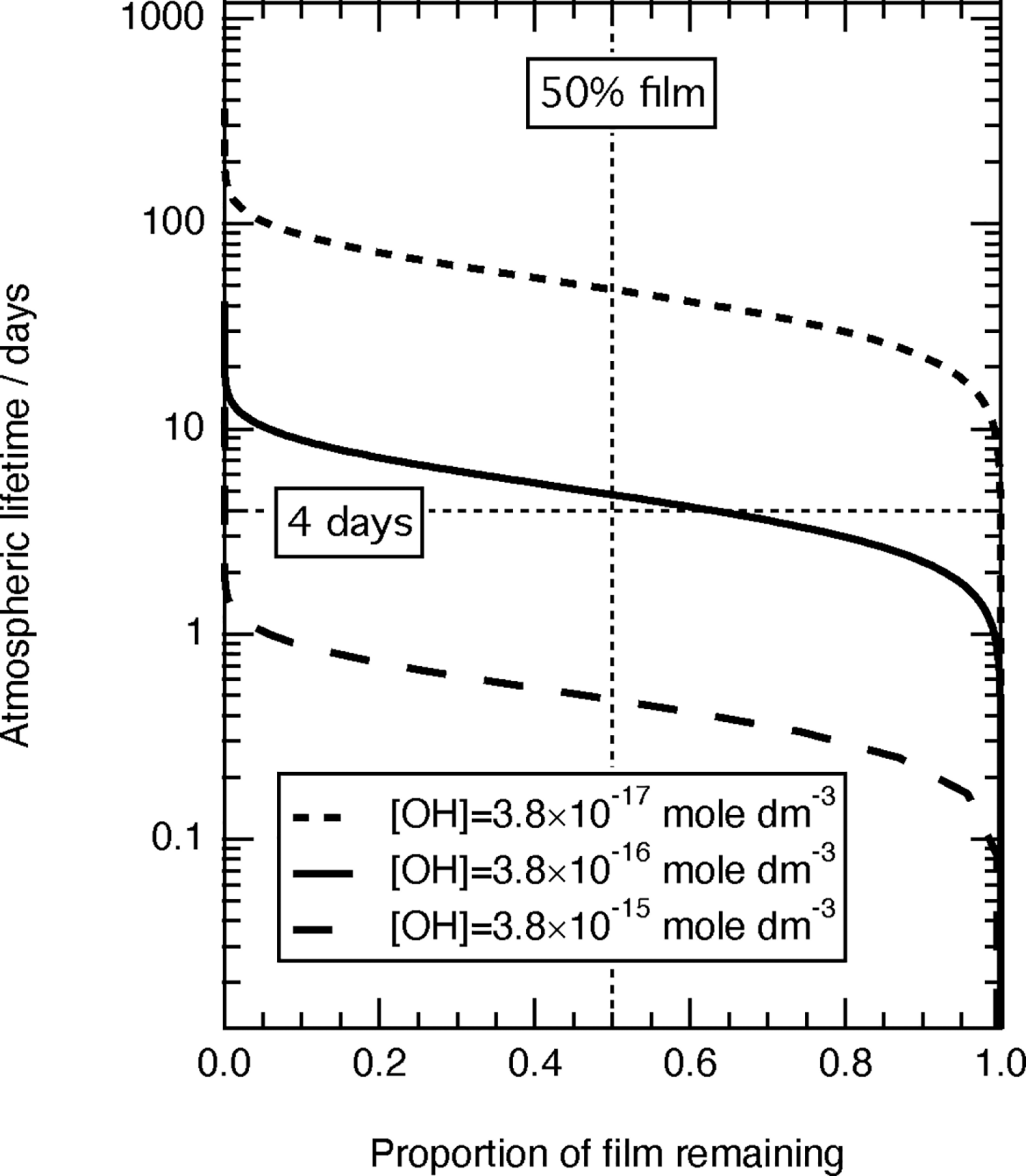
Atmospheric lifetime of the film versus proportion of the film remaining for three different ^•^OH radical concentrations. The vertical dashed line highlights the atmospheric half-life. The importance of the ^•^OH concentration is emphasized in the figure by considering that a typical aerosol lifetime is on the order of a few days.

The differential equations used to describe the degradation mechanism were solved analytically (see part 3 of Supporting Information) and the relative amount of the film at the air–solution interface with time was estimated using the bimolecular rate constants listed in Table 1 and ^•^OH concentrations of 3.80 × 10^–17^ mol dm^–3^, 3.80 × 10^–16^ mol dm^–3^,^103^ and 3.80 × 10^–15^ mol dm^–3^ to provide an envelope of film lifetimes. These ^•^OH concentrations bracket the range of atmospheric ^•^OH (aq) concentrations determined in the literature,^104−107^ and the estimated film lifetime is sensitive to these values. The estimated film lifetimes are shown in Figure 5, and the dotted vertical line corresponds to an estimate of the film half-life. Note that the film lifetime is different from the natural lifetime calculation in eq R49 (the characteristic time for the amount of DSPC to fall to 1/*e* of its initial value).

The solid black line in Figure 5 (^•^OH concentration, 3.80 × 10^–16^ mol dm^–3^) indicates that it takes ∼4–5 days for the amount of film material to decay to half of its initial amount, which is of the same order of magnitude as a typical aqueous aerosol lifetime and therefore suggests that oxidation initiated by the ^•^OH radical may be important to film lifetime in the atmosphere. The vertical dashed line emphasizes the film half-lives and indicates that the oxidation of the film depends on the ^•^OH radical concentration. An ^•^OH concentration of 3.80 × 10^–15^ mol dm^–3^ gives a half-life of ∼1/2 days whereas an ^•^OH concentration of 3.80 × 10^–17^ mol dm^–3^ gives a half-life greater than 50 days which is atmospherically unimportant. Thus the atmospheric importance of aqueous ^•^OH oxidation is very sensitive to ^•^OH concentrations in particles in the atmosphere. Few studies of ambient ^•^OH in droplets have been conducted.^103^

## Conclusions

The amount of deuterated material at the air–solution interface was observed to decay when a monolayer film of the fully deuterated lipid DSPC was exposed to the aqueous phase radicals ^•^OH, SO_4_^•–^, and NO_3_^•^. The decay was consistent with a multistep degradation mechanism with some products remaining at the air–solution interface that were subsequently oxidized by the aqueous radicals. Thus, lipid films on atmospheric aerosol will require several radical reaction steps to remove all organic material from the interface and atmospheric aqueous phase oxidation of lipid films at the air–solution interface can be described by multistep kinetics.

An atmospheric lifetime of ∼20 h was calculated for a DSPC molecule with respect to attack by an aqueous phase hydroxyl radical using a typical atmospheric concentration of aqueous hydroxyl radicals. The overall film lifetime was determined to have a half-life of ∼4–5 days, calculated using the literature concentration of 3.80 × 10^–16^ mol dm^–3^ for aqueous hydroxyl radicals.^103^ Oxidation of thin films in the atmosphere by aqueous radicals may therefore be important, and reactions of lipid films appear consistent with a multistep degradation mechanism.

## Supporting Information Available

Neutron reflectometry experiments conducted, e-folding depth, calculated steady state concentrations for thehydroxyl radical and sulfate anion, uncertainty in steady state hydroxyl radical concentration, sensitivity study of the number of steps and weighting constants used in the degradation kinetic modeling, and analytical solutions to the concentration–time profiles for the degradation mechanism used (PDF)
