# Supplementary material for: Aqueous Radical Initiated Oxidation of an Organic Monolayer at the Air–Water Interface as a Proxy for Thin Films on Atmospheric Aerosol Studied with Neutron Reflectometry
Source: J Phys Chem A. 2023 Oct 13;127(42):8922–34. doi: 10.1021/acs.jpca.3c03846 (PMC10614302; doi:10.1021/acs.jpca.3c03846)
Supplement: Supplementary file 1 — jp3c03846_si_001.pdf [file jp3c03846_si_001.pdf]

Electronic Supporting Information for "Aqueous Radical Initiated Oxidation of an Organic Monolayer at the Air-Water Interface as a Proxy for Thin Films on Atmospheric Aerosol Studied with Neutron Reflectometry"

Stephanie H. Jones [a,b †]*, *Martin D. King* [a]*, *Adrian R. Rennie* [c], *Andrew D. Ward* [b], *Richard A. Campbell* [d ‡] and *Arwel V. Hughes* [e]

[a] Centre of Climate, Ocean and Atmosphere, Department of Earth Sciences, Royal Holloway University of London, Egham, Surrey, TW20 0EX, U.K.
[b] STFC, Central Laser Facility, Research Complex at Harwell, Rutherford Appleton Laboratory, Harwell Oxford, Didcot, Oxfordshire, OX11 0FA, U.K.
[c] Department of Chemistry–Angström Laboratory, Uppsala University, 75121 Uppsala, Sweden.
[d] Institut Laue-Langevin, BP 156, 6, 71 avenue des Martyrs, CS 20156, F-38042 Grenoble Cedex 9, France.
[e] ISIS Pulsed Neutron and Muon source, Rutherford Appleton Laboratory, Harwell Oxford, Oxfordshire, OX11 0QX, U.K.

Present address

† Karlsruhe Institute of Technology (KIT), Institute of Meteorology and Climate Research, Atmospheric Aerosol Research Department, Hermann-von-Helmholtz-Platz 1, 76344 Eggenstein-Leopoldshafen, Germany.
‡ Division of Pharmacy & Optometry, Faculty of Medicine, Biology & Health, University of Manchester, Oxford Road, Manchester, M13 9PT, U.K.

Part 1

Table S1 Neutron reflectometry experiments conducted

Run Number	$\Gamma_{initial}/10^{18}$ molecule m^{-2}	Radical	Subphase pH	k'_{23} / 10^{-4} s^{-1}	k'_{24} / 10^{-4} s^{-1}	k'_{25} / 10^{-4} s^{-1}	k'_{26} / 10^{-4} s^{-1}
1	1.70	$NO_3^{\cdot}$	4.7	4.4	2.2	2.2	2.2
2 dark control	1.64	$NO_3^{\cdot}$	4.7	-	-	-	-
3	1.65	$NO_3^{\cdot}$	4.7	1.1	1.0	1.0	1.0
3 photolysis control	1.68		-	-	-	-	-
4	1.52	$NO_3^{\cdot}$	4.7	2.0	1.8	1.8	1.8
5	1.62	$NO_3^{\cdot}$	5.5	2.1	2.0	2.0	2.0
6	1.60	$NO_3^{\cdot}$	5.5	1.4	1.3	1.3	1.3
7	1.63	$NO_3^{\cdot}$	5.5	2.2	2.06	2.0	2.0
8	1.64	$NO_3^{\cdot}$	5.5	2.4	2.2	2.2	2.2
9	1.63	$NO_3^{\cdot}$	5.5	1.6	1.5	1.5	1.5
10	1.74	$SO_4^{\cdot-}$	5.5	1.8	1.57	1.5	1.5
11	1.59	$SO_4^{\cdot-}$	5.5	2.2	1.8	1.8	1.83
12	1.62	$SO_4^{\cdot-}$	-	2.7	2.2	2.2	2.2

13	1.63	$SO_4^{\cdot-}$	-	2.7	2.2	2.2	2.2
14	1.64	$SO_4^{\cdot-}$	-	2.7	2.2	2.2	2.2
15<br>photolysis control	1.58		5.5	-	-	-	-
16<br>dark control	1.58	$SO_4^{\cdot-}$	-	-	-	-	-
17	1.59	$SO_4^{\cdot-}$	-	1.3	1.1	1.1	1.1
18	1.59	$^{\cdot}OH$	5.5	3.7	2.2	2.4	2.0
19<br>photolysis control	1.59		5.5	-	-	-	-
20	1.62	$^{\cdot}OH$	5.5	3.3	2.0	2.2	1.8

[a] Table of pseudo-first order rate constants for each neutron experiment conducted, including the initial values of surface coverage and the pH of the precursor solution. It was assumed that all neutron reflectivity was due to DSPC. Experiments 1-4 and 10-11 were conducted on FIGARO at the ILL and experiments 5-9 and 12-20 were conducted on SURF at ISIS.

e-folding depth

It was noted that UV radiation from the lamps could be absorbed by either water or the aqueous radical precursor solution at 254 or 360 nm and consequently attenuate the light with increasing trough depth causing a decreasing irradiance with depth profile. If this were the case, the radical steady-state concentration would mirror the decreasing irradiance-depth profile. In order to assess the effect of light attenuation with increasing trough depth, the depth for incident light to decay to ~37% (1/e), the e-folding depth, was calculated and values are shown below. Values are significantly greater than the trough depth and thus light attenuation was not an issue. The trough was made of PTFE and was a Lambertian reflector, aiding in uniform diffuse reflection to further reduce any depth irradiance profile. The liquid depth of the trough was ~3.5 mm and the height of the meniscus was ~0.5 to 1 mm.

Table S2 e-folding depth

Photolysis wavelength/ nm	Absorption coefficient of water[101,102]/× 10^{-5} cm^{-1}	e-folding depth in water/ cm	Radical precursor	Radical precursor absorption cross section[64,67,103]/ cm^2 $molecule^{-1}$	Concentration of precursor solution/ mol dm^{-3}	e-folding depth for solution/ cm
360	6.56	6620	NO_3^- and $S_2O_8^{2-}$	NO_3^- ~ 0<br>$S_2O_8^{2-}$ = 2.42×10^{-21} this work	0.1 and 0.03	23
360	6.56	6620	$S_2O_8^{2-}$	2.42×10^{-21} this work	0.03	23
254	44.9	968	NO_3^-	1.45×10^{-20}	0.03	4

[a] Absorption coefficients for water, absorption cross sections for radical precursors and concentrations used to calculate the values of e-folding depth for water and each radical precursor solution at the photolysis wavelengths. The calculated values for both water and radical precursor e-folding depths are significantly greater than the depth of the trough, ~3.5 mm and therefore light is effectively not attenuated through the solution in the trough.

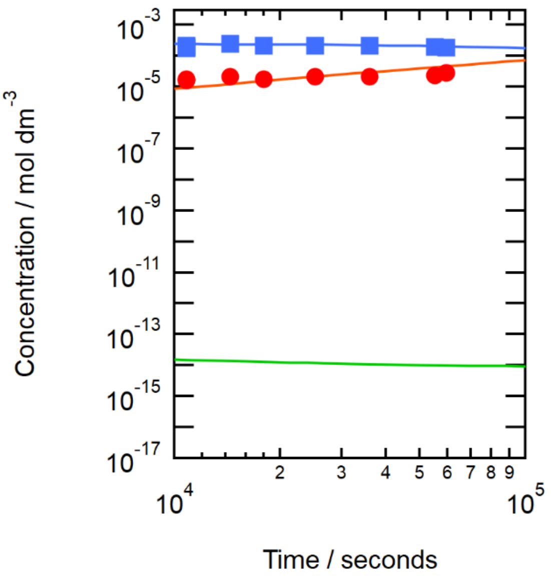

Figure S1. Experimental decay of NO_3^- anion (blue squares) and the production of NO_2^- anion (red circles) during photolysis of KNO_3. Kinetically modelled fits are shown as lines and the calculated steady-state hydroxyl radical concentration is the green line.

The experimental data points and kinetically modelled lines are for *exactly* the same experimental apparatus (trough, lamp, supporting scaffolding etc.) removed from the neutron beam and performed offline in similar laboratory with the same temperature. For the offline experiments, the concentration of the nitrate anion is 0.0003 M which is a factor of 100 lower than the experiment performed with a DSPC monolayer on the neutron facility beamline. A concentration of nitrate of 0.03 M does not appreciably change over the DSPC oxidation time and importantly the concentration of OH radical is not assumed to change. For the experiment plotted above the lower concentration is required so that the photolysis lamp makes a measurable change to the concentration of nitrate anion and so a kinetic model (reactions R4, R5, R9-R26) can be fitted to the temporal profile of the concentration of the nitrate and nitrite anions to enable the photolysis rate constants (reactions R4, R5 and R11) to be determined by variation until values that reproduce the experimental temporal nitrate and nitrite concentration profiles are found. The sensitivity to the fitting is demonstrated by increasing and decreasing the photolysis rate constants and is shown in Figure S3. The dashed lines in Figure S3 represent the temporal profiles calculated with variation of the photolysis rate constants. The dashed lines closest to the fit are ±20%, followed by ±40 % and so on for ±60 and ±80 %. The lines above the fit represent an increase in the photolysis rate constants and the lines below the fit represent a decrease in the photolysis rate constants. From this data we have chosen to say that the photolysis rate constants can be varied by ±40 % which corresponds to an uncertainty of ~± 30 % in the OH steady state concentration on the neutron beamline.

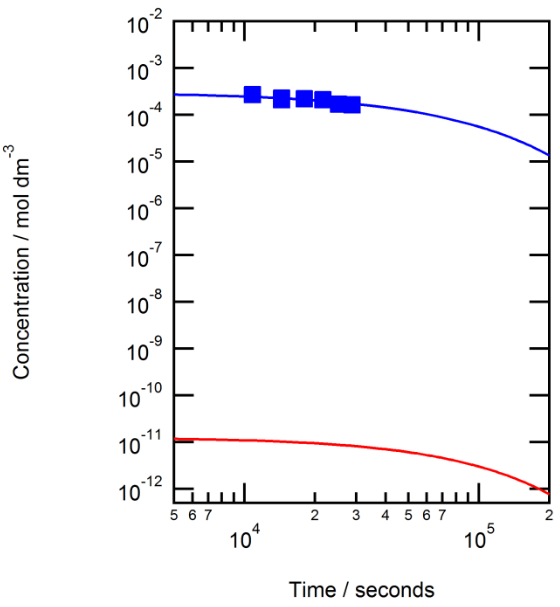

Figure S2. Experimental decay of the anion $S_2O_8^{2-}$ is shown as the blue squares during photolysis of $K_2S_2O_8$. The kinetically modelled fit is shown as the blue line. The resultant steady-state sulfate radical anion $SO_4^{\cdot-}$ concentration is shown as the red line.

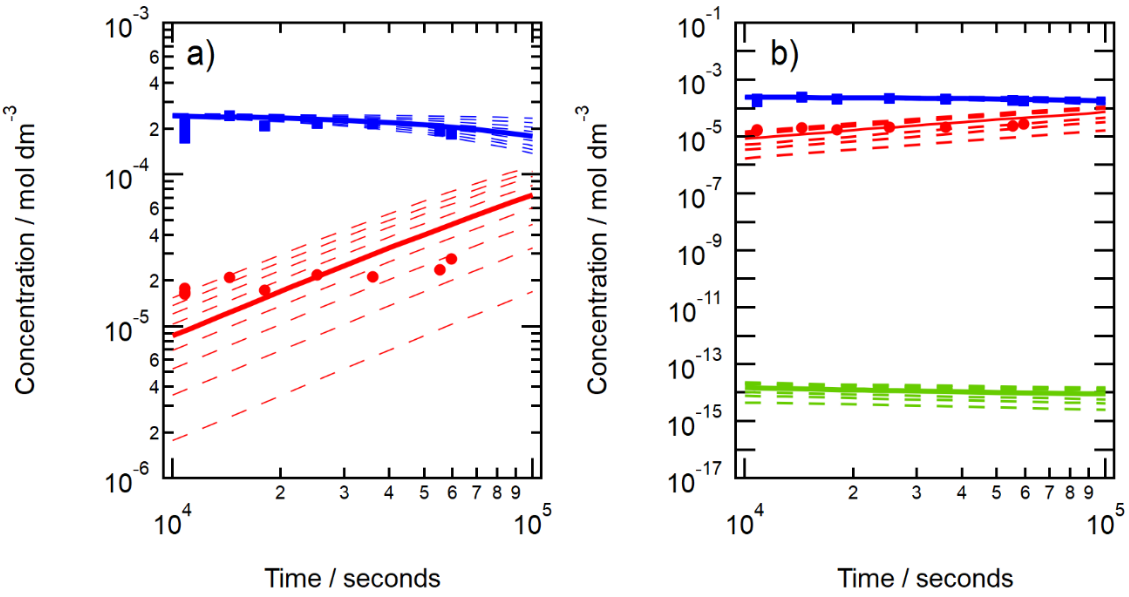

Figure S3. Measured offline concentrations for the nitrate anion (blue squares), nitrite anion (red circles) and the modelled steady-state OH concentration (solid green line in panel b) during photolysis of KNO_3. Solid lines show the model fits and dashed lines represent fits from variation of the photolysis rate constants. Panel a) shows the data and model fits for the nitrate

and nitrite concentrations and panel b) includes the calculated steady-state OH concentration. The dashed lines closest to the solid fit lines represent +20 % (above fit line) and –20 % (below fit line) variation in photolysis rate constants, the next dashed lines are ±40 % variation in photolysis rate constants and so on for ±60 % and ±80 %. From this analysis, ±40 % variation in the photolysis rate constants was deemed to give an acceptable model fit to the nitrate and nitrite concentrations which corresponds to a ~±30 % uncertainty in the steady-state OH concentration. Note the variation in steady-state OH concentration is determined using the same model but with the solution concentrations used in the neutron experiments i.e., 0.03 M, not 0.0003 M, used in the offline experiments.

Part 2. A sensitivity study of the number of steps and weighting constants used in the degradation kinetic modelling

In the main body of the paper the reactions below, are shown as needed to provide a good fit to the experimental data of $\frac{\rho\delta}{\rho_0\delta_0}$ versus reaction time, t:

$DSPC_{(surf)} + OH_{(aq)} \rightarrow A_{(surf)}$ (1)

$A_{(surf)} + OH_{(aq)} \rightarrow B_{(surf)}$ (2)

$B_{(surf)} + OH_{(aq)} \rightarrow C_{(surf)}$ (3)

$C_{(surf)} + OH_{(aq)} \rightarrow D_{(aq)}$ (4)

The fitting of the kinetic equations (1-4) to the decay of $\frac{\rho\delta}{\rho_0\delta_0}$ versus time, t, appears at first sight to have a large number of fitting variables and comparatively small number of data points to constrain the values of the fitted variables. Presented here is a description of the work to quantify the goodness-of-fit of the individual parameters k_1, k_2, k_3, k_4 and α, β, γ, and the number of individual reaction steps. It should be remembered that the purpose of fitting a kinetic chemical mechanism to the data is to demonstrate the decay is consistent with a degradation mechanism (i.e. repeated reaction of several OH radicals with the lipid DSPC until the reaction products are no longer surface active and partition to the air or water bulk phases). To demonstrate our fitting process is robust the goodness-of-fit is measured with the metric, χ^2, defined as,

$$\chi^2 = \sum_{i=0}^{n} \frac{(O_i - M_i)^2}{\sigma^2}$$

where O is the observed data point and M is the modelled data point and σ is the measure of uncertainty in the observed data point O. A small value of χ^2 is indicative of a good fit between the modelled kinetic decay and experimental kinetic decay. The examples given here will be for the reaction between the aqueous OH radical and DSPC. The demonstration here is equally applicable to reactions with the sulfate and nitrate radical but not displayed to save space and tedium.

The sensitivity of the values of the rate constants, k_1, k_2, k_3, k_4.

Figure S4 demonstrates the minima in metric for goodness of fit, χ^2, for the values of k_1, k_2, k_3, k_4. What is immediately obvious is that the minima are deep and unique.

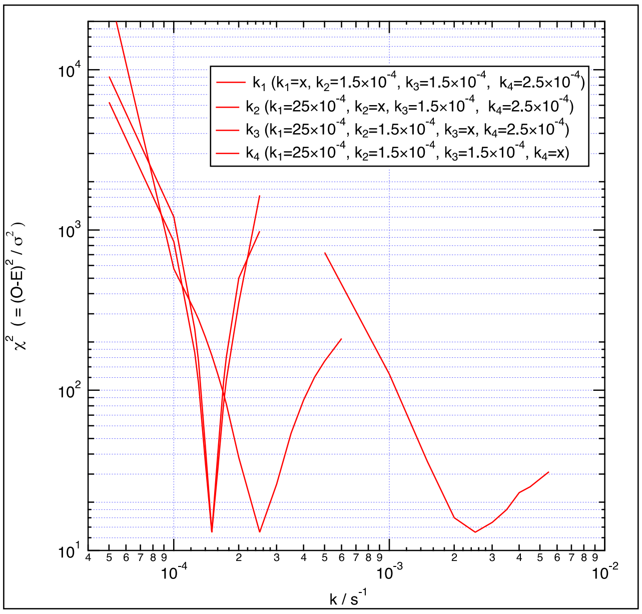

Figure S4. exploration of the goodness-of-fit minima by variation of the individual rate constants k_1, k_2, k_3, k_4.

It was found that the values of k_1, k_2, k_3, k_4. are not correlated and it is difficult to compensate for the reduction of one rate constant with the increase in another. To demonstrate this further Figure S5 shows the sensitivity of the modelled value to increasing and decreasing the value of k_1, k_2, k_3, k_4. by constant factors.

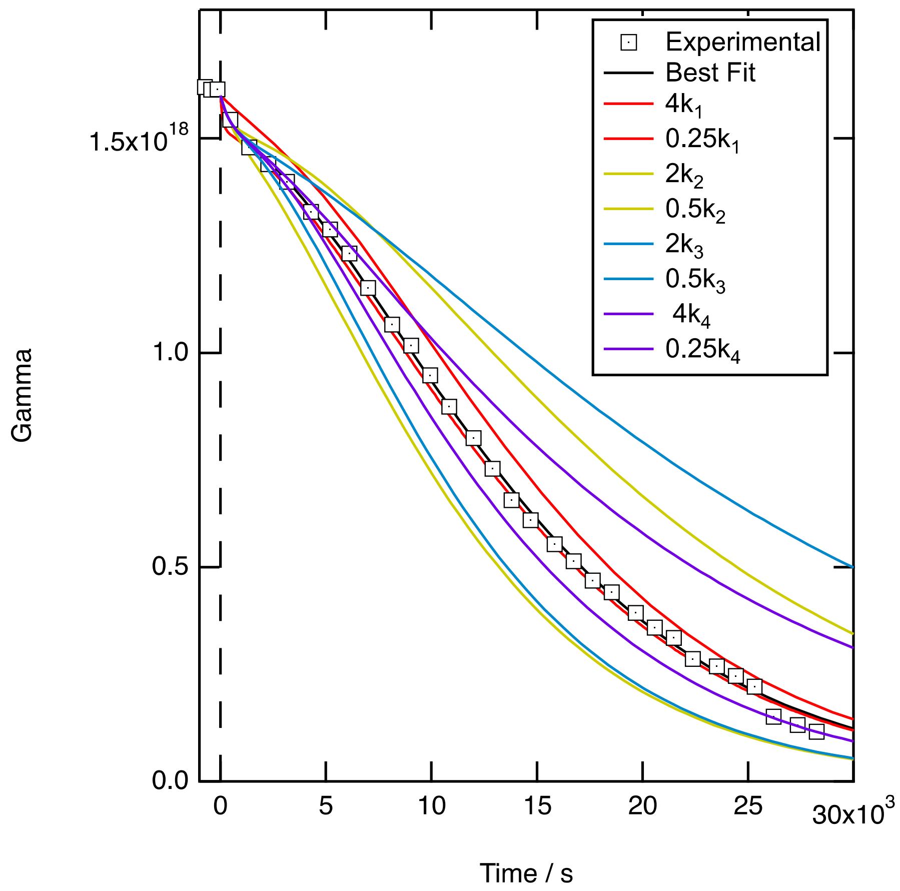

Figure S5. A sensitivity analysis of value of k_1, k_2, k_3, k_4. and its effect on reaction profile i.e.

$\Gamma = \frac{\rho\delta}{\rho_0\delta_0}$ versus time, t

As can be seen in Figure S5. Altering the value of the rate constant k_1, k_2, k_3, k_4 changes the shape of the reaction temporal profile in a distinctive manner that cannot be compensated by changing the value of another of k_1, k_2, k_3, k_4.

The sensitivity of the weightings α,β,γ.

Figure S6 demonstrates the robustness of the weighting factors equation R35. The values of the weighting factors, α, β, or γ, were varied, and the rate constants k_1, k_2, k_3, k_4 were optimized to gain the best fit by producing the lowest value of χ^2. The results are shown in Figure S6. It is immediately obvious that values of α, β, or γ, reported in the paper produce the lowest value of χ^2 and the rate constant changed to produce a good fit is not particularly sensitive to the value of α, β, or γ. It was found that the decrease in the metric of fit between experimental and modelled data points caused by individually changing the value of α, β, or γ, could be partially compensated for by varying one rate constant.

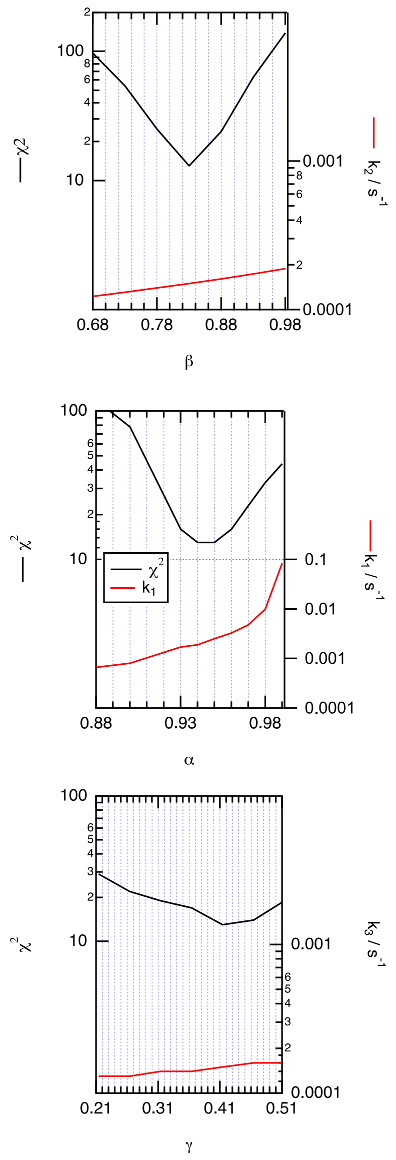

Figure S6. The variation of the weighting factors with the metric of goodness of fit.

The number of reaction steps.

Figure S7 justifies the decision of four reactions producing three surface active products A, B and C: The value of χ^2 decreases with the number of reactions producing surface active product until a value of three reactions is reached. The addition of another reaction producing four reactions with surface active products does not improve the goodness of fit and so was not used. Note the values of rate constants, k_1, k_2, k_3, k_4. were varied to get the best fit (a minimum value of χ^2) for each number of steps.

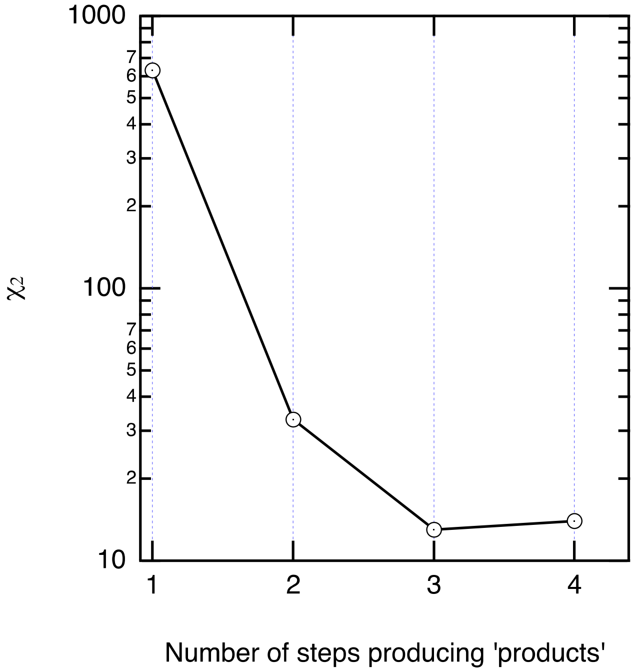

Figure S7. The lowest value of the metric of goodness-of-fit versus the number of reactions producing a surface-active product. More than three reactions producing a surface-active product does not improve the fit.

Part 3: Analytical solutions to the concentration-time profiles for the degradation mechanism used

The multi-step degradation mechanism,

$$\boldsymbol{DPSC_{(surf)} \longrightarrow A_{(surf)} \longrightarrow B_{(surf)} \longrightarrow C_{(surf)} \longrightarrow D_{(aq)}}$$

can be described by the following differential equations:

$$\frac{-d[DSPC]}{dt} = k'_1[DSPC]$$

$$\frac{d[A]}{dt} = k'_1[DSPC] - k'_2[A]$$

$$\frac{d[B]}{dt} = k'_2[A] - k'_3[B]$$

$$\frac{d[C]}{dt} = k'_3[B] - k'_4[C]$$

where k'_1, k'_2, k'_3 *and* k'_4 are pseudo first-order rate constants, i.e. $k'_1 = k_1[OH], k'_2 = k_2[OH], k'_3 = k_3[OH]$ *and* $k'_4 = k_4[OH]$. An integrating factor was used to solve the differential equations and the following solutions were obtained:

$$[DSPC] = [DSPC]_0 e^{-k'_1 t}$$

$$[A] = \frac{k'_1[DSPC]_0}{(k'_2 - k'_1)}(e^{-k'_1 t} - e^{-k'_2 t})$$

$$[B] = \frac{k'_1 k'_2[DSPC]_0}{(k'_2 - k'_1)}\left(\frac{e^{-k'_1 t}}{(k'_3 - k'_1)} - \frac{e^{-k'_2 t}}{(k'_3 - k'_1)} - \frac{e^{-k'_3 t}}{(k'_3 - k'_1)} + \frac{e^{-k'_4 t}}{k'_3 - k'_1)}\right)$$

$$[\boldsymbol{C}] = \frac{k_1' k_2' k_3' [DSPC]_0}{(k_2' - k_1')}\left(\frac{e^{-k_1' t}}{(k_3' - k_1')(k_4' - k_1')} - \frac{e^{-k_2' t}}{(k_3' - k_2')(k_4' - k_2')} - \frac{e^{-k_3' t}}{(k_3' - k_1')(k_4' - k_3')} + \frac{e^{-k_3' t}}{(k_3' - k_2')(k_4' - k_3')} - \frac{e^{-k_4' t}}{(k_3' - k_1')(k_4' - k_1')} + \frac{e^{-k_4' t}}{(k_3' - k_2')(k_4' - k_2')} + \frac{e^{-k_4' t}}{(k_3' - k_1')(k_4' - k_3')} - \frac{e^{-k_4' t}}{(k_3' - k_2')(k_4' - k_3')}\right)$$
